# Hypertension and its management in COVID-19 patients: The assorted view

**DOI:** 10.1016/j.ijcrp.2021.200121

**Published:** 2021-11-13

**Authors:** Gaber El-Saber Batiha, Abdulrahim Gari, Norhan Elshony, Hazem M. Shaheen, Murtala Bello Abubakar, Sherif Babatunde Adeyemi, Hayder M. Al-kuraishy

**Affiliations:** a Department of Pharmacology and Therapeutics, Faculty of Veterinary Medicine, Damanhour University, Damanhour, 22511, AlBeheira, Egypt; b Department of Obstetrics & Gynecology, Faculty of Medicine, Umm-Al-Qura University, Makkah, Saudi Arabia; c Obstetrics & Gynecology Dept, King Faisal Specialist Hospital and Research Center, Jeddah, Saudi Arabia; d Department of Physiology, Faculty of Basic Medical Sciences, College of Health Sciences, Usmanu Danfodiyo University, PMB 2254, Sokoto, Nigeria; e Centre for Advanced Medical Research and Training, Usmanu Danfodiyo University, PMB 2254, Sokoto, Nigeria; f Department of Plant Biology, Faculty of Life Sciences, University of Ilorin, PMB 1515, Ilorin, Nigeria; g CG Bhakta Institute of Biotechnology, Uka Tarsadia University, Gopal Vidyanagar, Bardoli-Mahuva Road, Tarsadi, Surat, 394350, Gujarat, India; h Department of Clinical Pharmacology and Therapeutic Medicine, College of Medicine, AL-mustansiriyiah University, Baghdad, Iraq

**Keywords:** SARS-CoV-2, Coronavirus disease 2019, Hypertension, ACE2

## Abstract

**Background:**

Coronavirus disease 2019 (COVID-19) is suspected to mainly be more deleterious in patients with underlying cardiovascular diseases (CVD). There is a strong association between hypertension and COVID-19 severity. The binding of SARS-CoV-2 to the angiotensin-converting enzyme 2 (ACE2) leads to deregulation of the renin-angiotensin-aldosterone system (RAAS) through down-regulation of ACE2 with subsequent increment of the harmful Ang II serum levels and reduction of the protective Ang-(1–7). Both angiotensin receptor blockers (ARBs) and angiotensin-converting enzyme inhibitors (ACEIs) are commonly used to manage hypertension.

**Objective:**

Objective was to illustrate the potential link between hypertension and COVID-19 regarding the role of angiotensin receptor blockers (ARBs) and angiotensin-converting enzyme inhibitors (ACEIs) in hypertensive patients with COVID-19.

**Methods:**

We carried out comprehensive databases search from late December 2019 to early January 2021 by using online engines of Web of Science, Research gate, Scopus, Google Scholar, and PubMed for published and preprinted articles.

**Results:**

The present study's findings showed that hypertension is regarded as an independent risk factor for COVID-19 severity. Both ACEIs and ARBs are beneficial in managing hypertensive patients.

**Conclusion:**

This study concluded that hypertension increases COVID-19 severity due to underlying endothelial dysfunctions and coagulopathy. COVID-19 might augment the hypertensive complications due to down-regulation of ACE2. The use of ACEIs or ARBs might be beneficial in the management of hypertensive patients with COVID-19.

## 1 Introduction

The world is currently experiencing a severe pandemic of severe acute respiratory syndrome coronavirus 2 (SARS-CoV-2) infections, which cause Coronavirus disease 2019 (COVID-19) [1]. It was observed that the mortality risk increases steadily in hypertensive patients who are positive for SARS-CoV-2, as observed in a meta-analysis of patients with underlying cardiovascular (CV) disorders, particularly hypertension, who are prone to the highest morbidity (10.5%) following infection [2]. It has been shown that patients with systemic hypertension are associated with a severe form of COVID-19 [3]. Patients with pre-existing cardiovascular disease are also at an increased risk of disease severity and death from COVID-19. Multiple cardiovascular complications have been associated with SARS-CoV-2 infection, including myocarditis, acute cardiac damage, arrhythmias, and thromboembolic diseases [2].

SARS-CoV-2 enters host cells via angiotensin-converting enzyme 2 (ACE2). ACE2 expression is enhanced in hypertension due to the renin-angiotensin system blockers that are frequently prescribed to hypertensive patients. These characteristics were hypothesized to be crucial in the infection and progression of COVID-19 [4].

Therefore, this review illustrates the relationship between COVID-19 and hypertension regarding angiotensin-converting enzyme inhibitors (ACEI) and angiotensin receptor blockers (ARBs).

## 2 Methods and search strategy

**Fig. 1:**
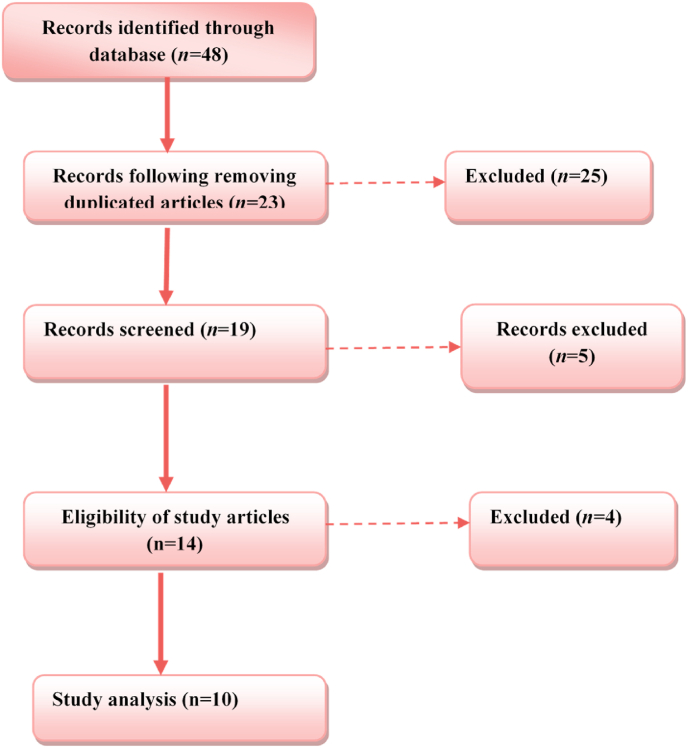
Flow-chart showing method and search strategy.

The current review was performed based on the Statement of Preferred Reporting Items for Systematic Review and Meta-Analysis (PRISMA). A comprehensive databases search was done from late December 2019 to early January 2021 by using online engines of Web of Science, Research gate, Scopus, Google Scholar, and PubMed for published and preprinted articles. We excluded duplicated papers, screened the data, and displayed the full-text documents (Fig. 1). Moreover, the quality of evidence was measured using the Grading of Recommendations Assessment, Development and Evaluation (GRADE) rating system [5], with evidence being graded as Grade A: Evidence usually derived from high-quality original studies. Grade B: Evidence derived from expert opinion as shown in Table 1.

**Table 1:** Available evidence summarises hypertension and COVID-19 findings derived from original studies and statements based on the expert opinion following the GRADE rating system.

Study conclusions	Evidence grade	Ref.
ACEI/ARBs increase ACE2 receptor expression; SARS-CoV-2 might utilise this increase to result in severe disease	A	[6]
Severely ill male patients with heart injury, hyperglycemia, and high-dose corticosteroid use may have a higher risk of death	B	[7]
The use of ACEI and/or ARBs can increase the risk of severity of COVID-19	A	[8]
Comorbidities such as COPD, diabetes, hypertension, and malignancy predispose individuals with COVID-19 to adverse clinical outcomes	A	[9]
Does not support discontinuation of ACEI/ARB medications that are clinically indicated in the context of the COVID-19 pandemic	B	[10]
A significant difference in the use of ACEI/ARB among patients with different severities of the disease	B	[11]
ACEI/ARBs reduce IL-6 and increase CD3 and CD8, thus reducing COVID-19 severity; ACEI and ARBs are beneficial in COVID-19	A	[12]
AT1R blockers, including ARBs, can help reduce COVID-19 morbidity and mortality	A	[13]
Animal data: increasing ACE2 expression can help protect against pulmonary and cardiovascular hazards; recommend continuing the use of ACEI and ARBs to manage hypertension in COVID-19 patients	A	[14]
RAAS inhibitors were shown to be possibly associated with a lower risk of mortality	B	[15]

## 3 Clinical presentation

**Fig. 2:**
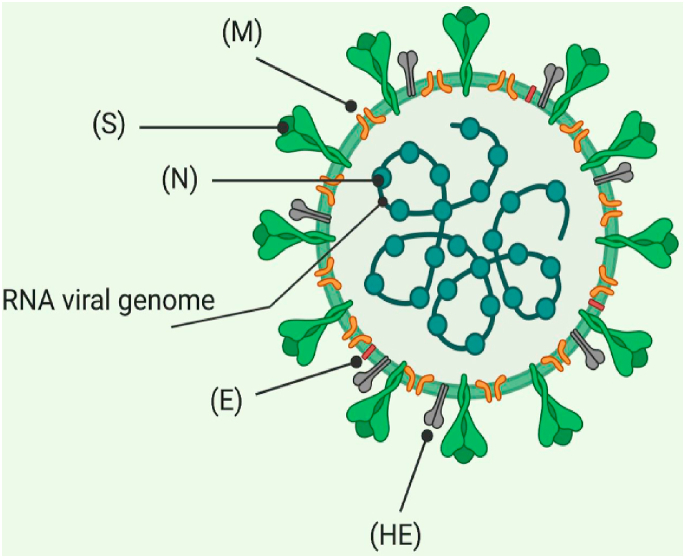
Structure of SARS-CoV-2. (M) Matrix, (S) spike glycoprotein, (N) N protein, (E) E protein, (HE) hemagglutination.

**Fig. 3:**
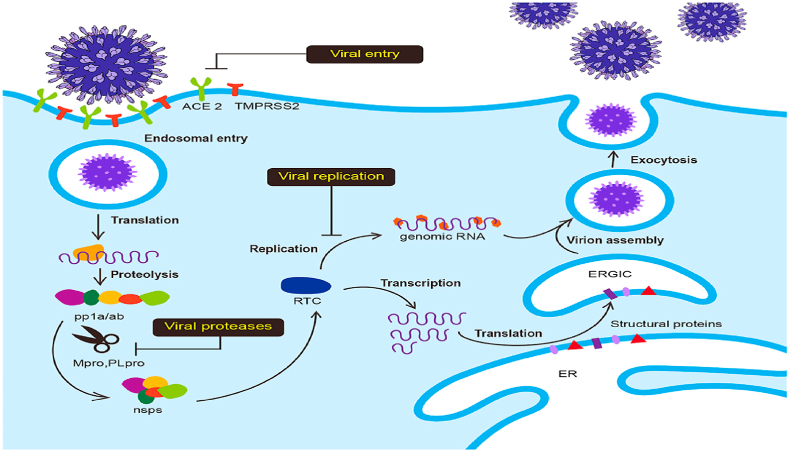
Coronavirus entry and replication inside the host cell. (1) Spike protein on the virion attaches to the cell-surface protein (ACE2), TMPRSS2, an enzyme that helps the virion to enter. (2) RNA is released by the virion. (3) Some RNAs are translated into proteins by the cell's machinery. (4) Formation of a replication complex by some of the proteins to make more RNAs. (5) Assembling of the proteins and RNAs into a new virion in the Golgi. (6) Release of new viruses.

The major features of SARS-CoV-2 are the higher degree of transmission together with an increased risk of death, mainly as a result development of acute respiratory distress syndrome (ARDS) [16]. SARS-CoV-2 (Fig. 2) is somewhat similar to the SARS-CoV that caused the SARS 2002 pandemic and shares some characteristics with the Middle East respiratory syndrome coronavirus (MERS-CoV) (MERS) [17]. The replication takes place through the RNA polymerase and encompasses the stoppage of transcription of mRNAs that encode six important open reading frames shared by all coronaviruses and many adjunct proteins (Fig. 3).

The typical presentation of COVID-19 are fever (87.9%), dry cough (67.7%), fatigue (38.1%), shortness of breath (18.6%), sore throat (13.9%), headache (13.6%), myalgia (14.8%), chills (11.4%), vomiting (5.0%), diarrhea (3.7%), hemoptysis (0.9%), and conjunctival congestion (0.8%) [18]. Patients with more severe infections are at risk of developing complications such as acute lung injury (ALI), acute respiratory distress syndrome (ARDS), and multi-organ failure [19].

## 4 COVID-19 and angiotensin-converting enzyme 2 (ACE2)

ACE2 is the chief regulator of the renin-angiotensin-aldosterone system (RAAS) and has a crucial role in regulating blood pressure [20]. ACE2 catalyzes the conversion of angiotensin II to angiotensin-(1–7), hence inactivating Ang II and its function in inflammation, oxidative stress, vasoconstriction, and fibrosis [21]. As well, ACE2 protects against several diseases, including hypertension and diabetes (through the protection of pancreatic β-cells) and other CVD, through down-regulation of Ang II [21].

**Fig. 4:**
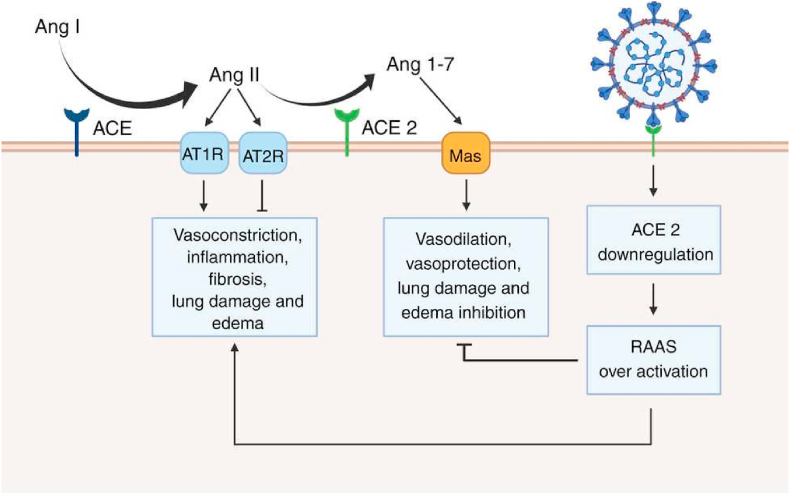
**Renin-angiotensin-aldosterone system (RAAS) in SARS-CoV-2 infection.** AngI is converted to AngII by the action of the angiotensin-converting enzyme (ACE). AngII acts angiotensin receptor type 1(AT1R), leads to vasoconstriction, inflammation, fibrosis, lung damage, and oedema. However, the action of AngII on the AT2R produces a reverse effect. ACE2 converts AngII to Ang1-7, which acts on the Mas receptor leading to vasodilation and lung protection. SARS-CoV-2 causes down-regulation of ACE2 with subsequent activation of RAAS, which causes dual effects.

SARS-CoV-2 requires ACE2 protein for cell entrance and activation of the spike protein (SP), including the receptor-binding region, which has a high affinity for the extracellular domain of ACE2 [22]. It is conceivable that SARS-CoV-2 internalization of ACE2 results in a loss of ACE2 activity on the cell surface, resulting in a decrease in Ang II degradation and an increase in Ang (1–7) synthesis [6]. Additionally, a higher level of Ang II has been observed in COVID-19 patients compared to apparently healthy subjects, which is thought to be associated with hypertension and pulmonary failure [23]. It is suspected that a strong correlation exists between decreased ACE2 activity and a bad prognosis in older COVID-19 patients. A deficiency of lung ACE2 may worsen systemic diseases, such as hypertension, due to induction of peripheral vasoconstriction, pro-inflammatory reactions, and oxidative stress (Fig. 4) [23,24].

## 5 COVID-19 and hypertension

Comorbidities, such as hypertension, have been reported to be significantly more frequent in COVID-19 patients treated in intensive care units (ICU) [9]. Hypertension potentially increases the patients’ susceptibility to developing a severe form of COVID-19 [25]. In COVID-19, circulating ACE2 receptors, which serve as the functional receptor for SARS-CoV-2, are increased. The presumed correlation between hypertension and COVID-19 may be linked to the deregulation of ACE2.

There is an increasing likelihood of admission of patients with pre-existing hypertension with SARS-CoV-2 infection compared to non-hypertensive patients [26]. Among hospitalized COVID-19 patients, the prevalence of hypertension and obesity is 50% and 48%, respectively. As well, the prevalence of underlying disorders recognized through COVID-NET was identical to those for hospitalized patients with influenza during 2018–2019, as 51% of patients had cardiovascular disorders excluding hypertension, 45% had chronic metabolic disorders, 40% had obesity, and 31% had chronic pulmonary diseases [27].

Several studies linked hypertension to poor prognosis in COVID-19. Based on ICU admission data, hypertension was the most prevalent comorbidity among 1382 COVID-19 patients, according to a meta-analysis and systematic review [28]. Bai et al.*,* [29] illustrated that critically ill COVID-19 patients are mostly hypertensive. In addition, a retrospective, multicenter-cohort study comprised all inpatients (>18 years old, n = 191) with COVID-19 from Jinyintan Hospital and Wuhan Pulmonary Hospital, who had been discharged with recovery or had died till January 31, 2020 showed that 137(71.73%) were discharged and 54(28.27%) died in hospital [7]. These data imply that hypertensive COVID-19 patients have a higher D-dimer level and persistent viral shedding. In Italy, the median age of COVID-19 patients who died was 79 years, and a more significant proportion (73%) were hypertensive. Additionally, 30% and 17% of patients who died were on ACEIs or ARBs, respectively [30]. In this consideration, there is a relatively small percentage of patients receiving these drugs despite the high percentage of individuals with hypertension and other cardiovascular diseases.

### 5.1 The link between COVID-19 and hypertension

The immunologic response, which is believed to be dysregulated in hypertension and SARS-CoV-2 infection, is one of the mechanisms linking hypertension and COVID-19 [9]. There are different inflammatory and metabolic disorders in SARS-CoV-2-related pathogenic mechanisms that alter the expression and function of ACE2 [31]. For instance, upregulation of ADAM metallopeptidase domain 17 (ADAM-17), a membrane-bound metalloprotease that contributes to the formation of tumour necrosis factor-α (TNFα), which works as a “shedder” by releasing attached receptors and pro-inflammatory cytokines [32]. The wild deterioration observed in COVID-19 patients is often linked to a burst in the circulating pro-inflammatory cytokines in COVID-19 patients [32].

Intriguingly, similar cytokines are also reported to be linked to the pathophysiology of hypertension in experimental studies [10]. For instance, IL-6, which has been strongly connected to clinical outcomes of COVID-19, appears to be a significant regulator of immunologic and inflammatory reactions in hypertension [33]. In pregnant rats, Orshal and Khalil [34] found that IL-6 enhanced vascular resistance and hypertension. It has been shown that hypomethylation of IL-6 is related to a higher risk of developing essential hypertension in matched case-control studies with patients and healthy controls of the same ages and gender [35]. These verdicts suggest that IL-6 is regarded as a pro-inflammatory cytokine that increases the risk for the development of hypertension.

In the COVID-19 era, IL-6 is regarded as one of the most important pro-inflammatory cytokines engaged with the development of cytokine storm and related complications, including ALI, ARDS and MOF [36]. IL-6 increases the risk for the development of COVID-19 severity and mortality by stimulating acute phase responses, specific immune reactions, and hematopoiesis [36]. A prospective cohort study involving 102 COVID-19 from Renmin Hospital, Wuhan, China, compared with 45 healthy controls, illustrated that IL-6 and other pro-inflammatory cytokines are higher in COVID-19 patients than controls [37]. Therefore, IL-6 serum level can be used as a predictor for rapid diagnosis of COVID-19 patients with a high risk of deterioration. Thus, IL-6 might be a surrogate biomarker in hypertensive COVID-19 patients.

A descriptive study involving 12 COVID-19 cases confirmed that lymphopenia could predict COVID-19 severity, poor clinical outcomes, and mortality. Also, lymphopenia in moderate COVID-19 was low at disease onset and higher than 20% at discharge time [38]. Overall, lymphopenia in hypertensive COVID-19 patients is considered a poor prognostic indicator. Furthermore, hypertensive individuals have deregulated CD4^+^ and CD8^+^ lymphocytes [39]. Similarly, immunosenescent CD8^+^ T cells failed to be activated during viral infections in hypertensive patients, which may explain COVID-19 severity in hypertensive patients [39]. It has been reported that adaptive and innate immune responses contribute to hypertension and associated MOF development. Various immune cell subpopulations, including B and T cells, NK cells, dendritic cells, and monocyte/macrophage, participate in vascular inflammation and induction of endothelial dysfunction in hypertension [37].

Harrison et al.*,* [40] divulged that antigen-presenting cells like macrophages and dendritic cells are intricate in presenting neo-antigens causing activation of T cells, which produce pro-inflammatory cytokines with successive development of hypertension. High pro-inflammatory cytokines trigger vasoconstriction, vascular remodeling, and sodium retention in the kidney [40]. Therefore, targeting of T cells activation and exaggerated immune response with attenuation release and effect of pro-inflammatory cytokines may reduce the severity of refractory hypertension.

In COVID-19, there is noteworthy activation of T and B cells with an exaggerated immune response that leads to severe complications due to higher release of pro-inflammatory cytokines with the development of cytokine storm [41]. Thus, it is proposed that abnormal immune response in COVID-19 could be a potential mechanism for developing overt hypertension and/or progression of pre-existing hypertension [42].

Moreover, COVID-19 patients with underlying comorbidities, including hypertension, are associated with decreased clearance of the SARS-CoV-2 virus [43]. Trump et al.*,* [44] observed that hypertension delays SARS-CoV-2 clearance and exacerbates lung inflammation in COVID-19 patients due to abnormal immune response and airway inflammation in hypertension. Therefore, hypertension by delaying SARS-CoV-2 clearance may increase COVID-19 severity and related complications.

On the other hand, there are controversies regarding the association between hypertension and COVID-19. Lippi et al.*,* [45] illustrated that hypertension significantly increases COVID-19 severity by about 2.5 fold. A meta-regression revealed that only old age groups with hypertension are associated with high COVID-19 severity. Suggesting that hypertension is weaker comorbidity increases COVID-19 severity following adjusting other confounding factors. Recent findings suggest no clinical evidence and scientific support for maintaining that hypertension or its treatment with ARBs/ACEIs contribute to critical outcomes in COVID-19 patients [46]. Of interest, the evidence of whether hypertension is a real independent risk factor in COVID-19 or not, so future researches is essential to clarify this multifaceted and complex puzzle.

Notably, elderly patients are at increased risk for COVID-19-related complications; ageing is also an established risk factor for developing hypertension. There is a progressive increase in the incidence and prevalence of hypertension to more than 60% in the elderly [47]. Therefore, it is sensible to predict that hypertension will be more frequent in COVID-19-related morbidity and mortality, as COVID-19 is severe and frequent in the elderly age group [48]. Indeed, COVID-19 comorbidities are self-reported at the time of admission, and many patients are not aware of their hypertensive condition. This may affect data interpretation and statistical results as supported by Wang et al.*,* [49] study that documented a lower prevalence of hypertension from 269 COVID-19 (16.9%), though the actual prevalence is 29.6%. Depending on these data, it is not clear whether hypertension is a potential prognostic factor in COVID-19. Therefore, inherent biases, age, and other anthropometric variables should be adjusted to elucidate the possible role of hypertension as an independent risk factor in COVID-19.

### 5.2 Obesity as a link for hypertension in COVID-19

It has been shown that there is a significant association between central obesity and hypertension, as increases follow typical overweight in blood pressure. A cross-sectional study involving 313,714 women aged 18 years or older illustrated that 32.8% of recruited patients had hypertension [50]. Moreover, obesity is a risk factor for developing severe COVID-19 due to underlying inflammatory cytokines linked with obesity that increase morbidity and mortality in SARS-CoV-2 infection [51]. Sattar et al.*,* [52] proposed that excessive fat deposition and obesity could be a potential risk factor for the development and progression of severe COVID-19 due to immune deregulation and impairment of the protective cardio-respiratory reserve that contributes to this progression of COVID-19-mediated complications.

In general, obesity is associated with an increased risk of cardio-metabolic complications, including hypertension, ischemic heart diseases, atrial fibrillation, heart failure, stroke, and diabetes mellitus [53,54]. Indeed, obesity provokes the development of endothelial dysfunction, prothrombotic status, and advancement of venous thromboembolism and disseminated intravascular coagulation that are hallmarks of severe COVID-19 [55,56]. Interestingly, obesity harms lung function, reducing vital capacity and forced expiratory volume leading to lower cardiorespiratory fitness and diminishes compensation during COVID-19 pneumonia [57]. Obesity is regarded as an inflammatory disease since; the adipose tissues, mainly in central obesity, release pro-inflammatory cytokines and adipokines, causing progression of immune-mediated diseases and metabolic disorders including hypertension and hypothyroidism [58]. Also, obesity and associated metabolic disturbances like hyperglycemia and hypertension impair the host defense mechanism against different viral infections, including COVID-19, with prolonged viral shedding time [59]. A retrospective longitudinal study comprised 126 COVID-19 with obesity showed a prolonged respiratory and fecal viral shedding due to abnormality in the CD3/CD56/NK cells axis [OR = 0.87. 95% CI = 0.76–0.99] that increases transmission and infectivity of SARS-CoV-2 infection [59].

Future considerations and narrative review by Cava et al.*,* [60] pointed to that obese COVID-19 patients are at higher risk for hospitalization and ICU admission for mechanical ventilation compared with lean subjects due to the underlying pro-inflammatory microenvironment, which aggravate the release of pro-inflammatory cytokines and development of cytokine storm in response to severe SARS-CoV-2 infection. Taken together, both obesity and hypertension are interrelated in the development of COVID-19 severity due to impairment of immune response, pro-inflammatory status, and coagulation/prothrombotic disturbances that trigger more severe complications in hypertensive COVID-19 patients with obesity [61].

In addition, obesity-related hypertension is due to activation of adipose tissue-tumor-necrosis-factor-alpha (TNF-α) and expression of angiotensinogen gene by nuclear factor kappa B (NF-κB) signaling pathway [62]. Therefore, NF-κB inhibitors can inhibit obesity-induced hypertension by mitigating adiposity-mediated hyper-inflammation and releasing proinflammatory cytokines implicated in the pathogenesis of hypertension [62]. Michalakis and colleagues illustrated that activated NF-κB signaling pathway in COVID-19 triggers release of IL-6, aldosterone and vascular endothelial growth factor (VEGF) from adipose tissue in obese patients with subsequent development of hypertension [63]. Likewise, activated NF-κB signaling pathway in SARS-CoV-2 can induce activation of node-like receptor pyrin 3 (NLRP3) inflammasome, which is also stimulated by SARS-CoV-2 ORF3a protein, leading to the release of pro-inflammatory cytokines, mainly IL-1β [64]. Besides, NLRP3 inflammasome is also activated in obesity and hypertension due to underlying low-grade inflammatory status [65,66].

These verdicts and findings suggest a potential role of obesity in developing hypertension in COVID-19 through induction of inflammatory signaling pathways and release of pro-inflammatory cytokines, which contribute to endothelial dysfunction and vascular resistance.

## 6 Role of ACEIs/ARBs in hypertensive COVID-19 patients

The RAAS involves various vasoactive peptides that organize essential physiological processes in humans and animals, and different studies suggest a significant association between RAAS and vulnerability to COVID-19. Therefore, modulation of RAAS by different agents and drugs may affect the outcome of COVID-19 patients since ACEIs and ARBs might have potential benefits in the management of hypertension, heart failure, and renal dysfunctions in COVID-19 patients [67]. There are underlying endothelial dysfunctions, pro-inflammatory disorders, and coagulopathy in hypertensive patients, which might increase COVID-19 severity [68]. Therefore, there is a mutual relationship between hypertension and the pathogenesis of SARS-CoV-2 infection.

Regarding the effect of antihypertensive drugs that modulate RAAS, such as ACEIs and ARBs, Yun et al.*,* [69] study involved 476 hypertensive COVID-19 patients showed that ARBs users illustrated a less severe form of COVID-19. However, no significant connection between the usage of ACEIs/ARBs and severe outcome or mortality from COVID-19 was discovered [70,71]. A retrospective review of medical reports and records from hospitalized COVID-19 patients admitted to the Shenzhen Hospital from 11 January to February 23, 2020 showed that COVID-19 patients receiving ARBs or ACEIs had a lesser disease severity and low level of IL-6 in peripheral blood [71]. Another study observed that COVID-19 patients with hypertension on ACEIs or ARBs treatments had a reduced frequency of disease severity and exhibited a tendency for reduced serum levels of IL-6 [72]. A meta-analysis included 33,483 COVID-19 patients from 11 studies illustrated no significant increment in the risk of SARS-CoV-2 infection [OR = 0.95, 95% CI = 0.89–1.05] in patients receiving ARBs/ACEIs therapy [72]. Based on current evidence, ARBs/ACEIs therapy should be continued in COVID-19 patients or those at risk to have COVID-19. This evidence supports the possible benefit of using ARBs or ACEIs in improving clinical outcomes in hypertensive COVID-19 patients.

Epidemiological studies report that about 30–40% of hypertensive patients diagnosed with COVID-19 in China were on ACEIs or ARBs alone or combined with other antihypertensive drugs in 25–30% of treated patients [49]. However, Ang-II levels were unchanged following treatment with early captopril therapy; nevertheless, with the administration of captopril monotherapy for six months [73].

This finding necessitated the hypothesis linking increased susceptibility to SARS-CoV-2 infection with pre-existing treatment with ACEIs or ARBs [74]. Indeed, despite Ang II, reported to promote the entry of ACE2 and intracellular degradation, losartan was found to reduce this effect, suggesting the possibility that ARBs may guard against the internalization of SARS-CoV-2 into the cells [75].

Regarding antihypertensive therapy mainly ACEIs/ARBs on the susceptibility for viral entry in COVID-19 concerning the role of ACE2 overexpression, five ongoing clinical trials test the safety of these drugs in COVID-19 patients. The PRAETORIAN- COVID trial, testing valsartan protective effect in COVID-19 patients without hypertension [76]. Of interest, ACEIs/ARBs upregulate ACE2, which is initially down-regulated during the acute phase of SARS-CoV-2 infection [63]. Therefore, ACEIs/ARBs have a protective role rather than harmful effect in COVID-19 patients.

Cohen et al.*,* [77] randomized elimination and prolongation of ACEIs/ARBs in COVID-19 Trial Protocol for illustrated continuation versus discontinuation of ACEIs/ARBs in COVID-19. REPLACE COVID depending on the primary and secondary outcomes, illustrated that ACEIs/ARBs may improve the host-defense mechanism and related inflammation, thereby reducing organ injury and giving direct protective effects. Besides, a BRACE-CORONA trial, which examined the outcomes of hospitalized COVID-19 patients randomized to continuation of ACEIs/ARBs or temporary suspension of these drugs. BRACE-CORONA trial involved 34 Brazilian medical sites that recruited 659 hypertensive COVID-19 patients and found that alive and out-of-hospital patients were identical between two groups [ RR = 0.97, 95% CI = 0.38–2.52, P = 0.95] [78]. This trial suggests and justifies no evidence implicating any role of ACEIs/ARBs with poor clinical outcomes in COVID-19.

Furthermore, ACEI-COVID, a prospective, randomized, parallel-group, controlled open-labeled trial, illustrated that discontinuation of ACEIs/ARBs or other RAS-inhibitors in COVID-19 had no significant effect on COVID-19 severity but led to better and faster recovery [79]. Taken together, REPLACE COVID, BRACE-CORONA, and ACEI-COVID trials give robust evidence for the beneficial effects of ACEIs/ARBs use in COVID-19. However, the decision to discontinue or continue ACEIs/ARBs should be made personally regarding the risk profile, indication, and availability of alternative treatments and outpatients’ monitoring options.

The immune system, mainly T-lymphocytes, contributes to hypertension aetiology, albeit the mechanism is unclear. Sympathetic stimulation may cause T-lymphocyte activation, aggravating vascular inflammation [80]. Thus, COVID-19-induced hypertension through induction of inflammation needs further researches and clinical studies [81]. Sun et al.*,* [82] reported that hypertension alone was not a precise, independent risk factor in predicting poor outcomes in COVID-19 patients. The multivariate logistic analysis illustrated that among 3400 COVID-19 patients with hypertension and/or diabetes, 3327(97.9%) survived, and 73(2.1%) died compared to COVID-19 patients without hypertension (n = 1392) [82]. Therefore, hypertension is not a potential risk factor in COVID-19, and the associated risk of hypertension is related to its confounding effect on diabetes mellitus.

Regarding ACE2 over-expression in COVID-19, theoretically, ACE2 facilitates the entrance of SARS-CoV-2 into the host cells, and higher expression of ACE2 may increase the severity of COVID-19 [83]. Pinto et al.*,* [84] observed that expression of ACE2 is augmented in COVID-19 patients with comorbidities, including hypertension, compared to the controls. However, there is no causal relationship between ACE2 over-expression and hypertension concerning the COVID-19 severity. It has been reported that ARBs upregulate ACE2 expression in an animal model study [85], though these finding has not been documented in the clinical settings concerning its role in COVID-19. One interesting study involving 112 COVID-19 patients with hypertension on ARBs illustrated that ARBs was not linked to morbidity and mortality [86]. Similarly, a retrospective analysis comprised 1128 hypertensive COVID-19 patients on ACEIs/ARBs treatments (n = 188) compared with those not taken this treatment (n = 940), following adjusting for gender, age, in-hospital medications, and other comorbidities showed that hypertensive COVID-19 patients on ACEIs/ARBs treatments had associated with low mortality compared to the other antihypertensive medications [9].

Additionally, an observational database of 8910 COVID-19 patients admitted to various hospitals in various countries indicated that underlying cardiovascular illnesses, but not hypertension, were associated with increased hospital mortality. However, there was no correlation between the usage of ACEIs/ARBs and in-hospital mortality [87]. Finally, Fernandez-Ruiz [88] observed that using ACEIs/ARBs is not associated with an increased risk of SARS-CoV-2 infection and has no adverse effect on COVID-19 patients. These findings imply that treating hypertensive COVID-19 patients with ACEIs/ARBs has a neutral or perhaps beneficial effect.

In a Japanese longitudinal cohort study of hypertensive patients, long-term olmesartan medication resulted in higher urinary ACE2 concentrations than untreated control patients, but not with enalapril or other ARBs such as losartan, candesartan, valsartan, or telmisartan [12]. The expression of ACE2 may also be up-regulated by the peroxisome proliferator-activated receptor-γ (PPAR-ɣ) and rosiglitazone (a PPAR-ɣ agonist), which have also been demonstrated to increase the expression of ACE2 [89]. Ciavarella et al.*,* [90] illustrated that PPAR-ɣ agonist modulates expression of ACE2 and reduces risk of cytokine storm in COVID-19.

Zhang et al.*,* [91] observed that telmisartan, an AT1R blocker, also exerts stimulation in PPAR-ɣ pathway leading to anti-inflammatory and antihypertensive effects. Similarly, telmisartan exerts anti-inflammatory and anti-atherosclerotic effects and improves endothelial dysfunction by stimulating PPAR-ɣ pathway in the macrophages [92]. A recent study revealed that telmisartan is regarded as a dual PPAR-ɣ/α agonist reducing hepatic inflammation and associated oxidative stress [93]. According to Heffernan et al.*,* [94], SARS-CoV-2 infection affects the PPAR-pathway, resulting in abnormal lipid metabolism in lung epithelial cells. This abnormal lipid metabolism contributes to lipotoxicity and inflammation, while also enhancing the infectivity and assembly of SARS-CoV-2. Thus, a PPAR- ɣ agonists such as fenofibrate or a PPAR−/−agonist combo such as telmisartan may be an option for COVID-19 patients with hypertension and metabolic abnormalities. Thus, by modifying ACE2 and inflammatory signalling pathways with various drugs such as ACEIs and ARBs, the risk of COVID-19 severity may be reduced.

This study has many limitations due to the observational nature of the research and their inherent biases. Numerous research compared hypertension with ICU admission in COVID-19 patients, limiting the scope of meta-analyses. The research also did not give an authoritative and thorough definition of hypertension. Similarly, little adjustments were made for confounders' risk factors, such as age and chronic comorbidities. Because most of the research works included were conducted and executed in China, it is difficult to rule out the importance of differences between nations. Additionally, understanding indicators of ICU admission may aid in the early and late phases of the COVID-19 pandemic's ICU and clinical treatment. Nonetheless, our findings are considered preliminary, and additional research is needed to corroborate our findings and conclusions.

The present study hypothesized that hypertension contributes to a complex effect with other comorbidities on mortality in COVID-19 patients.

## 7 Conclusion

This study concluded that hypertension increases COVID-19 severity due to underlying endothelial dysfunctions and coagulopathy. COVID-19 might augment the hypertensive complications due to down-regulation of ACE2. The use of ACEIs or ARBs might be beneficial in the management of hypertensive patients with COVID-19. Nonetheless, more robust clinical trials and prospective studies are imperative to confirm this beneficial effect in this regard.

## Funding disclosure

This work was not supported by any fund.

## Declaration of competing interest

The authors declare no conflict of interests.
